# Strategies for Targeting Retroviral Integration for Safer Gene Therapy: Advances and Challenges

**DOI:** 10.3389/fmolb.2021.662331

**Published:** 2021-05-12

**Authors:** Kristine E. Yoder, Anthony J. Rabe, Richard Fishel, Ross C. Larue

**Affiliations:** Department of Cancer Biology and Genetics, College of Medicine, The Ohio State University, Columbus, OH, United States

**Keywords:** retrovirus, gene therapy, targeted integration, HIV-1, MLV, LEDGF/p75

## Abstract

Retroviruses are obligate intracellular parasites that must integrate a copy of the viral genome into the host DNA. The integration reaction is performed by the viral enzyme integrase in complex with the two ends of the viral cDNA genome and yields an integrated provirus. Retroviral vector particles are attractive gene therapy delivery tools due to their stable integration. However, some retroviral integration events may dysregulate host oncogenes leading to cancer in gene therapy patients. Multiple strategies to target retroviral integration, particularly to genetic safe harbors, have been tested with limited success. Attempts to target integration may be limited by the multimerization of integrase or the presence of host co-factors for integration. Several retroviral integration complexes have evolved a mechanism of tethering to chromatin via a host protein. Integration host co-factors bind chromatin, anchoring the complex and allowing integration. The tethering factor allows for both close proximity to the target DNA and specificity of targeting. Each retrovirus appears to have distinct preferences for DNA sequence and chromatin features at the integration site. Tethering factors determine the preference for chromatin features, but do not affect the subtle sequence preference at the integration site. The sequence preference is likely intrinsic to the integrase protein. New developments may uncouple the requirement for a tethering factor and increase the ability to redirect retroviral integration.

## Introduction

**FIGURE 1:**
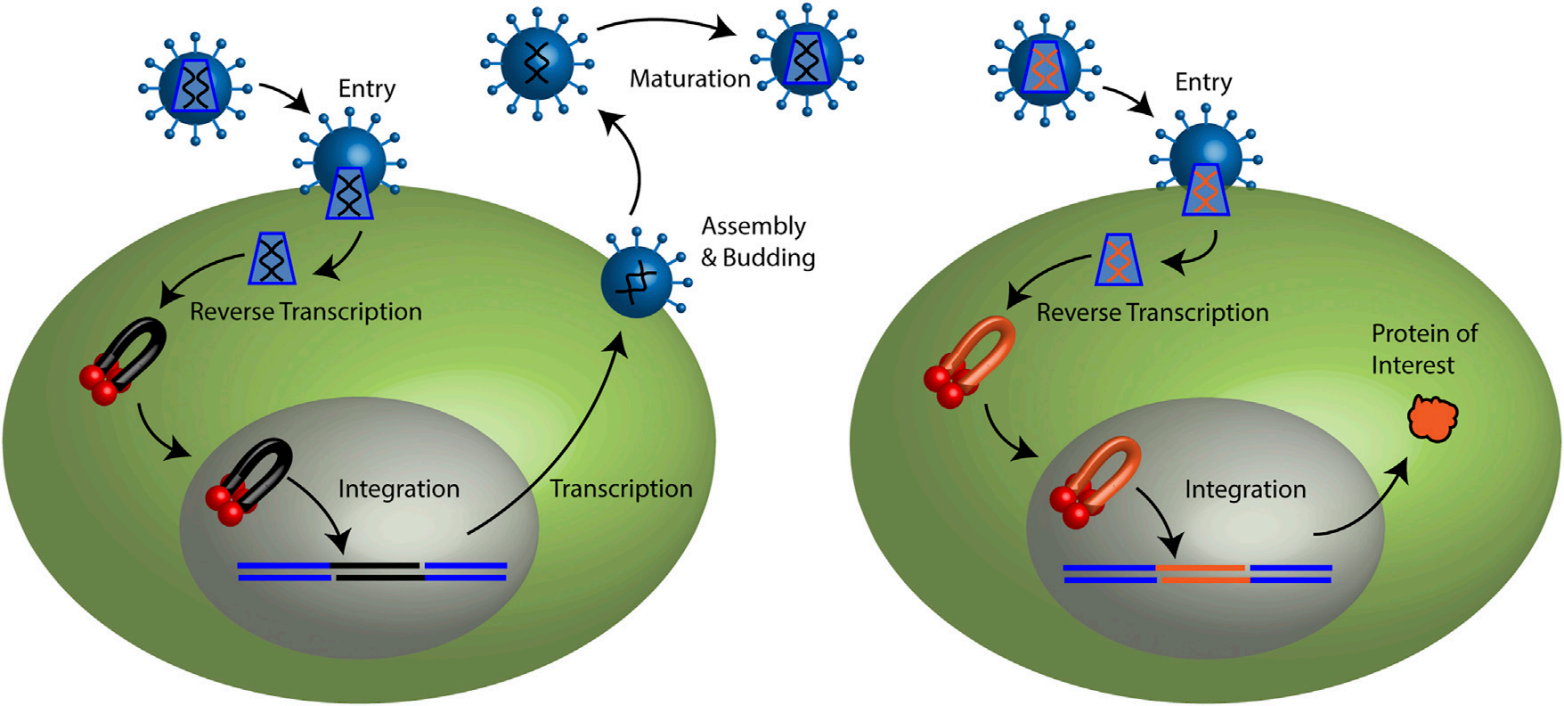
Retroviral life cycle and retroviral vector particle transduction. **(Left)** Retroviruses may enter a target cell by membrane fusion. The capsid core is released to the cytoplasm. Reverse transcription copies the viral genomic RNA (black lines) to a linear double stranded cDNA. Integrase binds the ends of the viral cDNA forming a pre-integration complex (PIC). Lentiviral PICs are able to cross an intact nuclear membrane while all other retroviruses require cellular division to access the host genome. Integrase mediates the stable integration of the vDNA (black) to the host genome (blue) generating the provirus. Host transcription machinery generates viral mRNAs and genomic RNA. Progeny viral particles assemble and are released from the plasma membrane. Following budding from the cell, viral enzyme protease cleaves the polyproteins to generate a mature infectious virus particle. **(Right)** Retroviral vector particles recapitulate the early steps of the retroviral life cycle. Viral RNA and cDNA depicted in red. However, they do not encode viral proteins. Only the protein of interest is expressed.

By stably inserting a transgene into a patient’s genome, retroviral gene therapy vectors offer the possibility of curing monogenic diseases (Sinn et al., 2005). Retroviruses are defined by the enzymatic activities of reverse transcriptase and integrase (IN) enzymes (Coffin et al., 1997). Reverse transcriptase copies the viral genomic RNA to a double stranded DNA (cDNA) (Figure 1). The nascent cDNA is bound by IN as part of a pre-integration complex (PIC). IN mediates the covalent joining of the viral cDNA ends to the host genome yielding the stably integrated provirus. Several families of retroviruses have been described including alpha (Rous sarcoma virus, RSV), beta (mouse mammary tumor virus, MMTV), gamma (murine leukemia virus, MLV), delta (human T cell leukemia virus, HTLV-1), epsilon (walleye dermal sarcoma virus, WDSV), lenti (human immunodeficiency virus, HIV-1), and spuma (prototype foamy virus, PFV). Retrovirus families alpha through epsilon are oncogenic in animals and humans. The lentiviruses cause immunodeficiency. The spumaviruses, also known as foamy viruses, have not been shown to cause any disease (Lindemann and Rethwilm, 2011).

There are several consequences of stable retroviral integration in patients. In the cases of human pathogens HTLV-1 and HIV-1, it has been impossible to cure patients with the notable exception of two HIV-1 patients (Taylor et al., 2019). During HIV-1 infection, a latent reservoir of cells persists throughout suppressive anti-retroviral therapy but will resume transcription and replication if therapy is stopped (Chun et al., 1999). Interestingly, some patients have been reported to suppress HIV-1 replication in the absence of anti-retroviral drugs (Kaul et al., 2011; Saez-Cirion et al., 2013; Sharaf et al., 2018). In most HTLV-1 patients the proviral genomes may remain transcriptionally silent, or latent, for decades (Bangham et al., 2019). Due to this prolonged latency, HTLV-1 infection does not lead to disease for 90–95% of patients. However, both diseases caused by HTLV-1, adult T cell leukemia (ATL) and neurodegenerative HTLV-1 associated myelopathy/tropical spastic paraparesis (HAM-TSP), have limited treatment options and poor prognoses (Utsunomiya et al., 2015; Yves et al., 2015; Matsuura et al., 2016).

**FIGURE 2:**
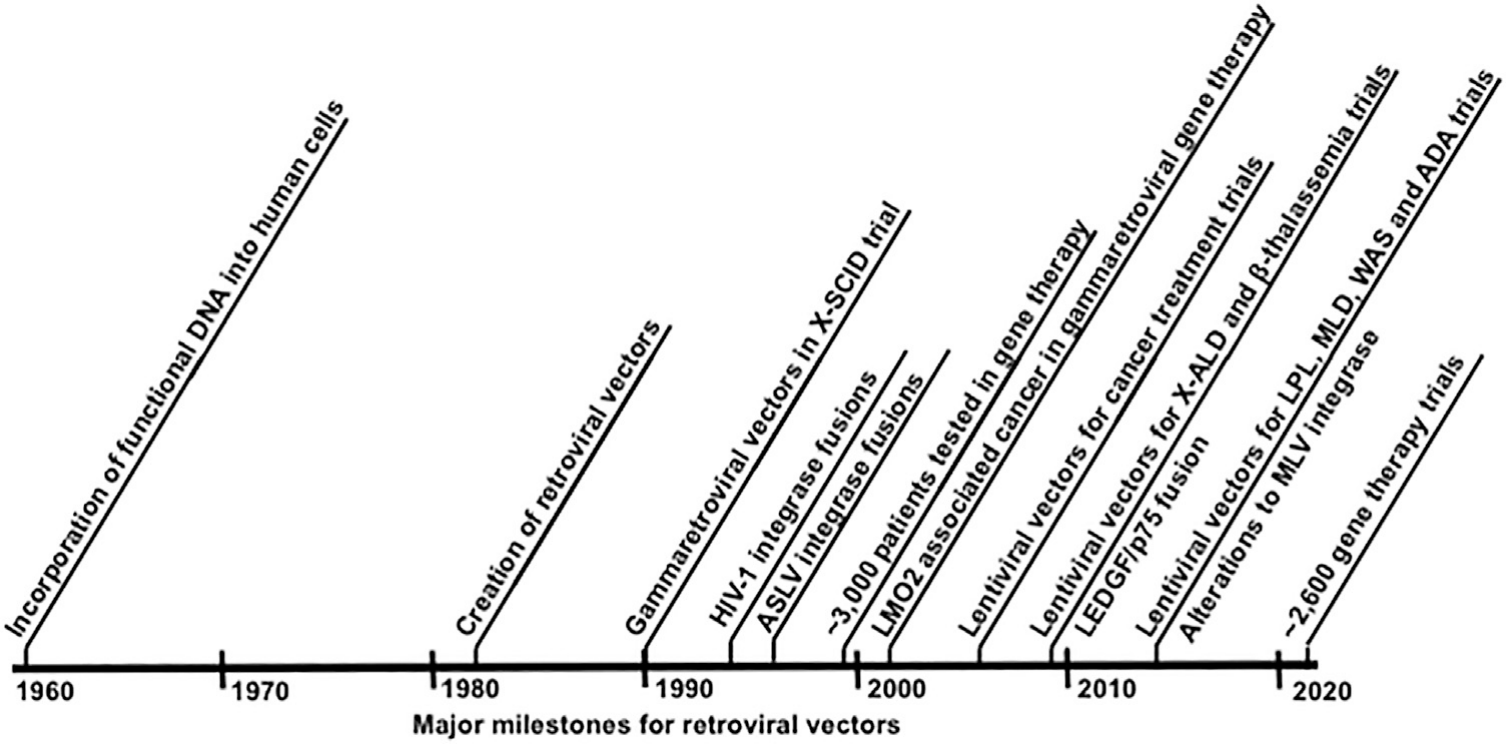
Timeline of developments in retroviral gene therapy vectors and targeted integration.

**FIGURE 3:**
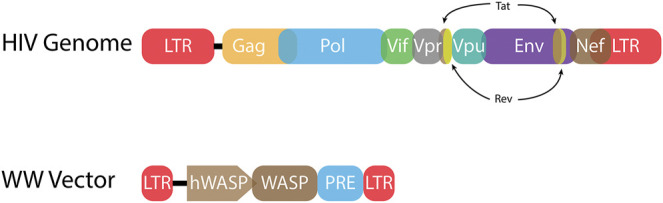
HIV-1 proviral genome and lentiviral vector genome. **(Top)** The HIV-1 proviral genome (HIV Genome) has long terminal repeats (LTRs) at each end. These non-coding sequences include the terminal sequences that are bound by integrase. The LTRs also encode sequences necessary for viral gene expression including transcription factor binding sites and a TATA box to initiate RNA Pol II transcription. Every retrovirus includes *gag, pol*, and *env* genes. These genes encode the structural, enzymatic, and envelope proteins, respectively. HIV-1 also has six accessory genes. Two of these genes, *tat* and *rev*, are spliced. **(Bottom)** A representative lentiviral vector (WW Vector) for treatment of Wiskott-Aldrich syndrome (WAS) encodes the *WAS protein* (*WASP*) gene driven by the human WASP promoter (hWASP) (Aiuti et al., 2013). The post-transcription regulatory element (PRE) mediates export of unspliced mRNA from the nucleus to the cytoplasm for translation (Zufferey et al., 1999). Much of the LTR sequences have been deleted, including transcription factor binding sites, yielding a self-inactivating (SIN) vector.

While the stable integration of retroviral genomes prevents effective cures of these viral infections, its very nature offers the exciting possibility to cure monogenic diseases with retroviral gene therapy vectors that stably introduce a corrective transgene (Table 1; Figure 2). Retroviral gene therapy vectors employ the viral structural and enzymatic proteins but lack accessory and/or virulence proteins (Miller 1992; Naldini et al., 1996; Trobridge et al., 2002a; Trobridge et al., 2002b). The packaged vector RNA genome encodes a cellular promotor, a corrective transgene, as well as regulatory elements required for packaging the RNA into vector particles (the psi packaging signal) and reverse transcription (the long terminal repeats) (Logan et al., 2002). No viral genes are encoded in the vector RNA genome (Figure 3). Retroviral particles are readily pseudotyped with a variety of membrane proteins altering the tropism and allowing entry to variable cell types (Duverge and Negroni 2020; Gutierrez-Guerrero et al., 2020). An advantage to retroviral-based vectors is the relatively limited induction of innate and adaptive immunity as compared to adenoviral-based gene therapy systems which have seen limitations due to their immunogenicity (Sauter and Kirchhoff 2016; Saez-Cirion and Manel 2018; Mennechet et al., 2019; Shirley et al., 2020). To date retroviral gene therapy vectors have allowed the delivery of functional transgenes to stem cells *ex vivo*, followed by successful engraftment and permanent functional cure of monogenic disorders. These disorders include X-linked chronic granulomatous disease (X-CGD), Wiskott-Aldrich syndrome (WAS), X-linked adrenoleukodystrophy, and X-linked severe combined immune deficiency (X-SCID) (Ott et al., 2006; Aiuti et al., 2013; Eichler et al., 2017). Despite this initial success, retroviral-based gene therapy vectors treating X-SCID suffered a significant setback when clinical studies with an MLV-based gene therapy vector led to leukemia in several children (Hacein-Bey-Abina et al., 2003; Hacein-Bey-Abina et al., 2008; Howe et al., 2008). Genetic characterization of the resulting cancer revealed that oncogenesis was due to MLV vector integration at the promoters of known oncogenes and dysregulation of their expression. Interestingly, this is also how MLV infection leads to leukemia in mice.

**TABLE 1:** Developments in retroviral gene therapy vectors.

	Advantages	Disadvantages	Advances	References
Gene therapy	Stable integration and expression of transgene	Oncogenesis	1983 - Creation of retroviral vectors	Perkins et al. (1983),Miller et al. (1983), Joyner and Bernstein, (1983)
	Low immunogenicity	Limited ability to target select genes	1990 - MLV vectors in patients for X-SCID	Blaese et al. (1995),Cavazzana-Calvo et al. (2000)
			2006 - HIV-1 vectors in patients for cancer treatment	Morgan et al. (2006),Bobisse et al. (2009),Johnson et al. (2009)
			2009 - HIV-1 vectors in patients for X-ALD and β-thalassemia	Cartier et al. (2009),Cavazzana-Calvo et al. (2010)
			2018 - CRISPR-CAS9 in patients	Romero et al. (2018)
Fusions of IN	Transduction of primary cells	Potential disruption of intasome multimers	1994 - First chimeric HIV-1 IN fusions	Bushman (1994),Goulaouic and Chow (1996)
	Modification of retroviral protein only	Reduced integration efficiency	1996 - First chimeric ASLV IN fusions	Katz et al. (1996)
			1997 - Zinc finger fusions to HIV-1 IN	Bushman and Miller (1997),Tan et al. (2004)
Fusions of tethering factors	Does not require modification of retroviral proteins	Cannot be performed in primary cells	2003 - Discovery of LEDGF/p75 as HIV-1 IN co-factor	Cherepanov et al. (2003),Turlure et al. (2004)
		Does not redirect all integration events	2009 - First LEDGF/p75 fusions	Meehan et al. (2009),Ferris et al. (2010),Silvers et al. (2010)
		Requires manipulation of cellular factors	2013 - Discovery of BET proteins as MLV IN co-factors	De Rijck et al. (2013),Gupta et al. (2013),Sharma et al. (2013)
			2013 - LEDGF/p75 fusion employed in WT cells	Vets et al. (2013)
Tether independent targeting	Transduction of primary cells	Limited efficacy	2016 - Alterations to PFV GAG	Hocum et al. (2016)
	No cellular modifications required		2014 - Alterations to MLV IN	Aiyer et al. (2014),Larue et al. (2014),El Ashkar et al. (2014)

Targeting retroviral integration to genetic “safe harbors” in the host genome that will not lead to cancer has become an imperative for the use of retroviral gene therapy vectors (Papapetrou et al., 2011). Integration site selection in a host genome is not random for most retroviruses (Schroder et al., 2002; Wu et al., 2003; Desfarges and Ciuffi, 2010; Serrao et al., 2015). Instead, each retrovirus displays unique integration preferences for genomic regions such as transcription units, CpG islands, or transcription start sites (TSSs). Understanding the factors governing species specific retroviral integration site selection is key for the development of next generation retroviral gene therapy vectors. While precision targeting of retroviral integration was first attempted over 25 years ago, thus far there has been little success in these endeavors. Whether retroviral gene therapy vectors can be purposefully directed to integrate at genetic safe harbors in patient cells is currently unknown.

Adeno-associated virus (AAV) vectors, which are not retroviruses, are also used as gene therapy vectors and may sometimes integrate at a specific locus in the human genome. AAV vectors are beyond the scope of this manuscript and have been extensively reviewed elsewhere (Wang et al., 2019; Colon-Thillet et al., 2021; Fakhiri and Grimm, 2021; Peters et al., 2021; Riyad and Weber, 2021).

### Integration Site Selection in Cells

**FIGURE 4:**
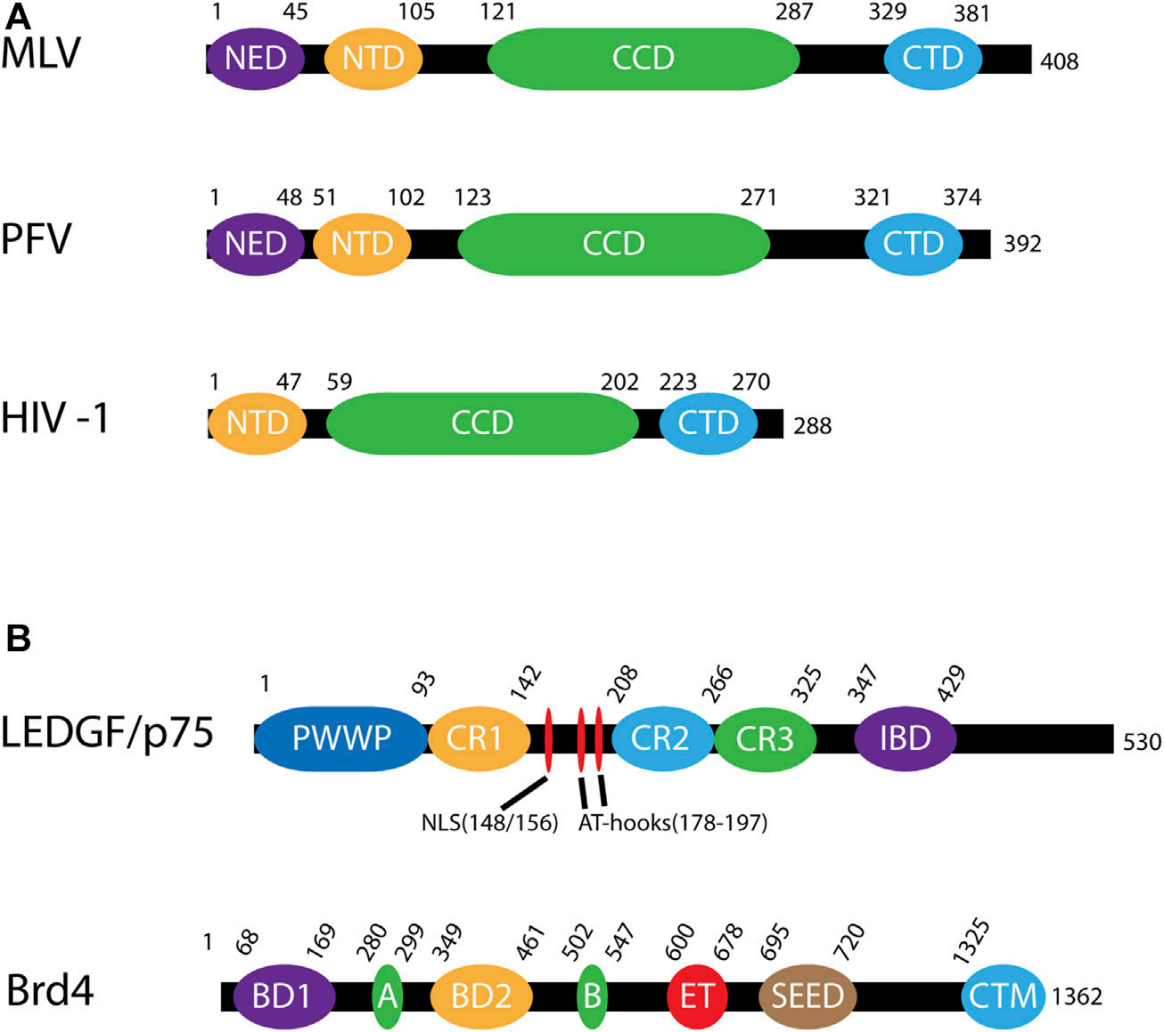
Domains of viral integrases and cofactors. **(A)** Domains of murine leukemia virus (MLV), prototype foamy virus (PFV) and human immunodeficiency virus (HIV-1) INs. These domains are the N-terminal extension domain (NED), the N-terminal domain (NTD), the catalytic core domain (CCD) and the C-terminal domain (CTD). The numbers along each line represent amino acid residues. **(B)** Domains of integration host cofactors LEDGF/p75 and Brd4. LEDGF/p75 includes a chromatin binding domain (PWWP) followed by three charged regions (CR1-CR3). Between CR1 and CR2 is a nuclear localization signal (NLS) and two AT-hooks which allow for DNA binding of AT-rich motifs. Near the C-terminus is the integrase binding domain (IBD). Brd4 contains two bromodomains (BD1, BD2) as well as 2 DNA binding motifs, A and B, which in conjunction bind chromatin. There is also a protein interaction extra terminal domain (ET), a serine-rich SEED domain, and a C-terminal motif (CTM).

Retroviral gene therapy vectors that have been used in humans and animals have been derived from MLV, HIV-1, avian sarcoma leukosis virus (ASLV), and PFV. All retroviral INs have a zinc coordinating amino terminal domain (NTD), a catalytic core domain (CCD) with the enzymatic DD (35)E motif, and a carboxyl terminal domain (CTD) that is the least conserved between retroviruses (Chiu and Davies, 2004; Figure 4A). Some retroviral INs, such as PFV IN, include an amino terminal extension domain (NED) (Valkov et al., 2009). Whether MLV IN includes a NED is controversial (Guan et al., 2017). Viral vectors derived from gammaretroviruses and spumaviruses, such as MLV and PFV, require cellular division to access the host genome while lentiviral vectors can traverse an intact nuclear membrane (Kobiler et al., 2012; Matreyek and Engelman, 2013). The ability of lentiviruses to infect non-dividing cells makes them especially attractive for gene therapy development.

Retroviral IN catalyzes two reactions during infection. Following reverse transcription of the viral genomic RNA to a linear double stranded cDNA IN removes a GT dinucleotide from the 3′-terminus of each viral DNA end, termed 3′-processing. In the nucleus IN covalently joins hydroxyls at both 3′ ends of the viral cDNA to the host DNA in independent single step transesterification reactions (Brown, 1997; Jones, Lopez et al., 2016). These two points of joining are separated by 4–6 base pairs of host DNA spanning one major groove. This spacing is characteristic of each retrovirus and results in duplications flanking the integrated proviral genome (Kvaratskhelia et al., 2014). Integration appears to be an inefficient reaction since only approximately 10% of reverse transcripts result in a provirus (Butler et al., 2001; Yoder et al., 2011; Francis et al., 2014; Francis and Melikyan, 2018).

Each retrovirus appears to have a distinct preference for integration site selection in cells (Shun et al., 2007). These preferences may favor or disfavor genomic elements, such as promoters or transcription units, or DNA sequence. While retroviral integration is not random in most cases, the preferences for chromatin elements are not stringent. For example, HIV-1 integration sites favor the bodies of actively transcribed genes (58–86% compared to a matched randomized control value of 45.7%) while murine leukemia virus (MLV) favors TSSs, enhancers, and promoter regions (15–39% compared to a matched randomized control value of 4.9%) with variations likely due to cell type and bioinformatics criteria (Schroder et al., 2002; Wu et al., 2003; Sharma et al., 2013; De Ravin et al., 2014; Lafave et al., 2014; Serrao et al., 2015; Feng et al., 2016). However, it should be noted that these retroviruses also integrate at sites outside of these regions (∼40–30% for HIV-1 integration not in actively transcribed genes and ∼70–80% for MLV integration outside promoter regions). Integration at genomic elements appears to be largely determined by host co-factors of integration (Shun et al., 2007).

In addition to genomic elements most retroviruses appear to have a unique subtle sequence preference at the points of joining (Holman and Coffin, 2005; Wu et al., 2005; Hacker et al., 2006; Kang et al., 2006; Marshall et al., 2007; Bennett et al., 2014; Liu et al., 2015). The preferences include the 4–6 bp between the points of joining and flanking 3 bp. The sequences appear to display palindromy, although this notion has been challenged (Kirk et al., 2016). The sequence preferences are extremely subtle generally requiring at least one hundred unique integration sites to achieve statistical significance at each base (Mitchell et al., 2004; Holman and Coffin, 2005; Wu et al., 2005; Bennett et al., 2014). There is no apparent linkage between any of the individual base preferences around the integration site. In addition, the consensus integration site preference is typically not observed. The DNA sequence preference at the integration site is unaffected by deletion of the host co-factors, suggesting it is determined by IN (reviewed in (Kvaratskhelia et al., 2014)).

Many retrovirus families have host proteins that act as integration co-factors (Cherepanov et al., 2003; De Rijck et al., 2013; Gupta et al., 2013; Sharma et al., 2013; Maertens, 2016; Winans et al., 2017). These proteins bind to both IN and chromatin effectively tethering the integration complex and directing integration to nearby host DNA. Tethering factors appear to determine the integration preference for genomic elements (Shun et al., 2007). To date two main groups of tethering factors have been identified: lens epithelium-derived growth factor (LEDGF/p75) is the host co-factor for lentiviral IN and the bromodomain and extra terminal (BET) family of proteins (Brd2, 3 and 4) interact with gammaretroviral IN.

**FIGURE 5:**
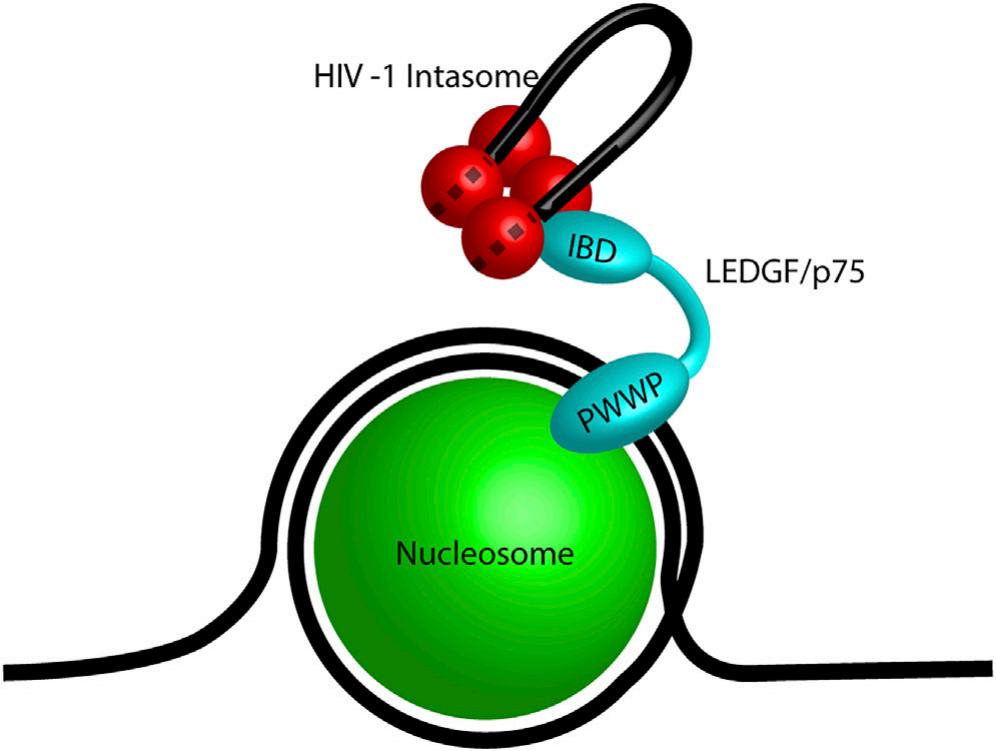
Model of LEDGF/p75 tethering an HIV-1 intasome. A mononucleosome (green) wrapped in DNA (black line) representing a nucleosome. The PWWP domain of LEDGF/p75 (cyan) binds the post translational modification H3K36me3. The integrase binding domain (IBD) of LEDGF/p75 is bound to the HIV-1 intasome (red) which is shown as a tetramer for simplicity. The viral DNA genome is shown in black and the dashed lines represent the viral DNA within the intasome.

The first identified retroviral integration host co-factor was LEDGF/p75 which interacts with HIV-1 IN (Cherepanov, Maertens et al., 2003; Turlure et al., 2004). LEDGF/p75 (encoded by *PSIP1*) is a transcriptional co-activator which interacts with a variety of cellular proteins including menin, mixed-lineage leukemia histone methyltransferase (MLL), and pogo transposable element with ZNF domain (PogZ) (Ge et al., 1998; Yokoyama and Cleary, 2008; Bartholomeeusen et al., 2009; Huang et al., 2012). It consists of a PWWP domain, three charged regions (CR), two AT hook domains, a nuclear localization signal (NLS), and an IN binding domain (IBD) (Figure 4B; Cherepanov et al., 2004; Llano et al., 2006a; Turlure et al., 2006). The PWWP domain of LEDGF/p75 binds to the histone H3 with a tri-methyl post translational modification (PTM) at lysine 36 (H3K36me3) (Pradeepa et al., 2012; Eidahl et al., 2013; van Nuland et al., 2013). H3K36me3 is a chromatin mark for active genes, the preferred target for HIV-1 integration (Schroder et al., 2002; De Rijck et al., 2010). The IBD binds to a cleft between two HIV-1 IN protomers and helps to stabilize the integration complex (Cherepanov et al., 2005; McKee et al., 2008; Kessl et al., 2011). The IBD domain is responsible for interactions with cellular proteins including cell division cycle-associated 7-like protein (JPO2), PogZ, and protein IWS1 homolog (IWS1) (Bartholomeeusen et al., 2009; Tesina et al., 2015). Taken together, LEDGF/p75 creates a bimodal tether between HIV-1 integration complexes and H3K36me3 in chromatin. These structural studies generated the model for a cellular factor guiding retroviral IN to a chromatin target site (Figure 5).

Genetic deletion of *PSIP1* reduced HIV-1 infection 10-fold and reduced integration into actively transcribed genes (Llano et al., 2004; Ciuffi et al., 2005; Shun et al., 2007). Sequencing HIV-1 integration sites in cells with deletion of the *PSIP1* gene revealed that the sequence preference was unaffected, suggesting that an IN tethering factor may not participate in the sequence preference (Shun et al., 2007). Ectopic expression of the IBD domain significantly inhibited integration by ∼7-fold (De Rijck et al., 2006; Llano et al., 2006a; Meehan et al., 2011). Small molecules that inhibit the interaction of LEDGF/p75 with HIV-1 IN (termed Allosteric IN inhibitors (ALLINIs), also referred to as non-catalytic site integrase inhibitors (NCINIs); LEDGINs or INLAIs) have been shown to alter HIV-1 infectivity, integration site selection, and virion maturation (Christ et al., 2012; Kessl et al., 2012; Tsiang et al., 2012; Feng et al., 2016). The primary mechanism of ALLINIs has been shown to be during virion maturation where it inhibits integrase interaction with the viral RNA genome, however ALLINIs exhibit secondary effects during integration via blocking integrase interaction with LEDGF/p75 (Jurado et al., 2013; Sharma et al., 2014; Kessl et al., 2016). Treatment with one such drug, BI-D, decreased HIV-1 integration in genes from 86.4% to 67.9% (Feng et al., 2016). Together these data indicate that LEDGF/p75 binding to HIV-1 IN directs integration to actively transcribed genes.

BET proteins are the principal binding partners of MLV IN (De Rijck et al., 2013; Gupta et al., 2013; Sharma et al., 2013). The BET protein family consists of Brd2, 3, 4, and T, whereas the extended BET family includes Brd1, 7, 8, and 9 (Wu and Chiang, 2007; Belkina and Denis, 2012). Brd2, 3 and 4 are ubiquitously expressed and have been implicated in control of the cell cycle, transcription, and DNA replication, whereas BrdT is only expressed in the testis. Brd4, unlike Brd2 and 3, is expressed in two isoforms: short (1–720) and long (1–1363) (Figure 4B; Boehm et al., 2012; Zhu et al., 2012). BET proteins are comprised of dual bromodomains (BD1 and BD2), DNA binding motifs A and B, and two C-terminal domains termed the extra terminal (ET) and SEED domain. The ET domain interacts with many proteins, including jumonji C-domain-containing protein 6 (JMJD6), histone-lysine N-methyltransferase NSD3 (NSD3), glioma tumor suppressor candidate region gene 1 protein (GLTSCR1), ATPase family AAA domain-containing protein 5 (ATAD5), and chromodomain helicase DNA-binding protein 4 (CHD4), as well as viral γ-2 herpesvirus latency-associated nuclear antigen (Rahman et al., 2011; Crowe et al., 2016). Of particular interest to this review is that the ET domain interacts with high affinity (160 nM) to the carboxyl terminal tail of MLV IN (Larue et al., 2014). The binding site in this highly flexible tail domain is conserved among gammaretroviruses but not other retroviral genera (Kvaratskhelia et al., 2014). Like LEDGF/p75 and HIV-1 integration, BET proteins direct MLV integration site selection through the bimodal association of its ET domain with the carboxyl terminal tail of MLV IN and its bromodomains with acetylated H3 and H4 histone tails (Moriniere et al., 2009; Filippakopoulos et al., 2010). The BET proteins guide MLV integration to TSSs, enhancers and super-enhancers (SE) (Sharma et al., 2013; De Ravin et al., 2014; Lafave et al., 2014). BET proteins play significant roles in human oncogenesis. Emerging anti-cancer small molecule inhibitors rely on acetylation mimics which block the BET bromodomains from binding chromatin (Alqahtani et al., 2019; Cochran et al., 2019; Zaware and Zhou, 2019; Lu et al., 2020; Perner and Armstrong, 2020). For example, archetypical compounds I-BET and JQ-1 were shown to bind the BET bromodomains and disrupt SE function in myeloid leukemia (Zuber et al., 2011; Loven et al., 2013). SE formation results in abnormally elevated expression of oncogenes and oncogenesis (Pelish et al., 2015; Call et al., 2020; Deng et al., 2020). During MLV infection, inhibition of BET proteins with JQ-1 significantly reduces integration at TSSs from 39 to 11% as well as corresponding reduction of integration near acetylated histone PTMs H3K9ac and H327ac (Sharma et al., 2013). Specifically, integration was reduced near oncogenes *LMO-2*, *CCND2*, and *BMI1*, the loci associated with tumorigenesis in human gene therapy trials (Larue et al., 2014).

Other retroviral IN tethering factors include the FACT complex and serine/threonine protein phosphatase 2A (PP2A). Alpharetrovirus ASLV IN has been shown to bind the heterodimeric facilitates chromatin transcription (FACT) complex which stimulates integration activity *in vitro* (Winans et al., 2017). Furthermore, depletion of the FACT complex in cells decreased ASLV integration efficiency. The FACT complex contains the structure specific recognition protein 1 (SSRP1) and suppressor of Ty 16 (Spt16), which form a general histone chaperone complex essential for transcription and DNA replication (Orphanides et al., 1998; Orphanides et al., 1999; Belotserkovskaya and Reinberg, 2004; Abe et al., 2011). While the FACT complex is thought to destabilize the histone octamer during cellular functions, such as DNA replication, its role in ASLV integration has not yet been determined (Reinberg and Sims, 2006; Winkler and Luger, 2011; Formosa, 2012; Winans et al., 2017). Deltaretroviral (HTLV-1, HTLV-2, and bovine leukemia virus) INs reportedly bind PP2A and its addition stimulated integration *in vitro* (Maertens, 2016). However, PP2A does not have any known chromatin binding function making its role during integration *in vivo* unclear.

Additional cellular factors have been shown to be involved in targeting retroviral integration to select genomic features. While not the focus of this review, a brief discussion is warranted. These factors have been studied in the context of their interaction with the HIV-1 capsid (CA) core and include cleavage and polyadenylation specificity factor subunit 6 (CPSF6), nucleoporin protein 153 (Nup153), and E3 SUMO-protein ligase (RANBP2 or Nup358). CPSF6 is a chromatin associated protein and a member of several nuclear complexes such as cleavage factor Im (CFIm) complex, paraspeckles, and nuclear speckles (Cardinale et al., 2007; Ruepp et al., 2009). It interacts with the HIV-1 CA core at the nuclear pore and the nuclear interior where it then directs the CA core/preintegration complex (PIC) toward gene dense regions (Lee et al., 2010; Price et al., 2012; Sowd et al., 2016; Achuthan et al., 2018; Bejarano et al., 2019). Unlike targeting by LEDGF/p75, which performs localized targeting within active genes, CPSF6 appears to target integration toward larger chromatin gene dense areas, potentially due to CPSF6 localization in nuclear speckles (Francis et al., 2020; Rensen et al., 2021). Nup153 is a component of the nuclear pore complex and serves as an essential scaffolding element (Krull et al., 2004). Nup153 interacts with the HIV-1 CA core similar to CPSF6, is essential for HIV-1 replication, helps to import the CA core/PIC into the nucleus, and its depletion retargets integration to less gene dense regions (Matreyek and Engelman, 2011; Koh et al., 2013; Matreyek et al., 2013; Lelek et al., 2015; Buffone et al., 2018). Nucleoporin protein RANBP2 interacts with the docking cytoplasmic CA core (Yokoyama et al., 1995; Frosst et al., 2002; Bichel et al., 2013). Like CPSF6 and Nup153, depletion of RANBP2 results in HIV-1 integration away from gene dense regions (Ocwieja et al., 2011). Taken together, the interaction of RANBP2 and Nup153 with the CA core encapsulating the PIC could explain the propensity for HIV-1 integration closer to the nuclear pore (Marini et al., 2015). However, retargeting integration of gene therapy vectors through alterations in HIV-1 CA appear unlikely due to the essential and genetically fragile nature of the CA core (Rihn et al., 2013). In addition, depletion of these additional cellular factors does not alter integration into active genes but instead retargets away from gene dense regions. Such broad retargeting is unlikely to aid in developing improved gene therapy vectors for integration at safe harbors.

### Retroviral Gene Therapy Vectors

Early animal and human studies using MLV-based gene therapy vectors were initially very promising. The first retroviral-based vectors were created in the early 1980s with other viral vectors such as those using adenovirus components following in the mid-1990s (Joyner and Bernstein, 1983; Miller et al., 1983; Perkins et al., 1983; Flotte et al., 1996). Murine bone marrow progenitor cells were transduced with MLV-based vectors expressing human *gp91* ^*phox*^ *ex vivo* and engrafted into mice with X-CGD. The mice displayed partial reconstitution of superoxide production, increased phagocytosis, and significantly increased survival after challenge with *B. cepacia* (Dinauer et al., 1999). In humans, MLV-based gene therapy vectors were first successfully used in hematopoietic stem cell (HSC) gene-therapy of X-SCID (Blaese et al., 1995; Cavazzana-Calvo et al., 2000). In clinical trials from 1999 to 2009, 20 X-SCID patients received gene therapy for a defect in the interleukin 2 common gamma chain gene encoding γ_c_ (also called IL-2RG (Wu and Dunbar, 2011)). While 17 patients were successfully treated for X-SCID, 5 of 20 patients developed leukemia (Wu and Dunbar, 2011; Cavazzana et al., 2016). These tumors correlated with integration of the MLV vectors near the *LMO-2* and *CCND2* oncogenes resulting in increased transcription (Schmidt et al., 2002; Hacein-Bey-Abina et al., 2003; Hacein-Bey-Abina et al., 2008). Patients who had received gene therapy for other genetic disorders, such as WAS and X-CGD, also developed leukemia (Dewey et al., 2006; Ott et al., 2006; Boztug et al., 2010; Stein et al., 2010). While these studies demonstrated the successful treatment of human genetic disorders with MLV-based vectors, they also revealed the significant hazards inherent to retroviral integration.

Following the development of leukemia in several MLV vector treated patients, the retroviral gene therapy field shifted focus to lentiviral-based vectors. Initially, there was concern that these vectors could also be oncogenic or have other unexpected deleterious outcomes. Lentiviruses lead to immunosuppression but are not oncogenic. Patients receiving anti-retroviral therapy (ART) do have higher incidence of non-AIDS-defining malignancies (NADM) such as Hodgkin’s lymphoma, oropharyngeal cancer, anal cancer, hepatocellular carcinoma, and non–small cell lung cancer (Lurain et al., 2019). Increased incidence of these malignancies could due to several reasons, including immunosuppression, co-infection with other oncogenic viruses such as Hepatitis B, and chronic immune activation and/or dysregulation (de Martel et al., 2015; Saeidi et al., 2018; Pinato et al., 2019). Studies evaluating links between HIV-1 infection and increased NADM have examined integration sites in cells and in cells from patients receiving ART. In HEK293T cells it was observed that HIV-1 integration events are ∼3-fold enriched in cancer driving genes and highly mutated genes (identified in the Cancer Genome Atlas) (Kandoth et al., 2013; Vogelstein et al., 2013; Singh et al., 2015). In patients receiving ART, it was observed that ∼40% of total integration events were detected in clonally expanded latent cells including integration into *MKL2* and *BACH2* oncogenes (Maldarelli et al., 2014). A second study showed similar results in HIV-1 repressed patients receiving ART with slightly enriched clonal expansion seen in oncogenes (Wagner et al., 2014). While these clonal expansions have been shown to play a role in reemergence of HIV-1 viremia in patients discontinuing ART, there has not been any validated link to cancer onset (Gantner et al., 2020; Halvas et al., 2020).

Lentiviral-based vectors are more amenable for manipulation in the clinic due to their ability to infect non-dividing cells (Swiggard et al., 2005). When cells are transduced in a resting, non-activated state, such as naïve T cells or HSCs, they may retain more functional potential when engrafted into patients (McLean and Michie, 1995). Thus, lentiviral-based vectors have been employed in multiple clinical trials, including for the treatment of certain blood cancers (Morgan et al., 2006; Bobisse et al., 2009; Johnson et al., 2009; Milone and O’Doherty, 2018). Development of clinically relevant lentiviral-based vectors has included removal of virulence factors, splitting the genome to multiple plasmids to reduce recombination, and deletions of viral promoter elements in the LTR to generate self-inactivating (SIN) vectors (Dull et al., 1998; Vannucci et al., 2013). In early generations of lentiviral-based vectors, insertional mutagenesis was observed in proliferative HSCs and tumors were observed in mice (Themis et al., 2005; Wu and Dunbar, 2011). Later generations of lentiviral-based vectors have successfully treated a variety of genetic diseases, such as WAS, metachromatic leukodystrophy, beta-thalassemia, X-linked adrenoleukodystrophy, and metachromatic leukodystrophy (Cartier et al., 2009; Aiuti et al., 2013; Biffi et al., 2013; Milone and O’Doherty, 2018). For example, gene therapy treatment of beta-thalassemia has led to several patients no longer requiring transfusions for several years post treatment, suggesting stable long-term expression and no apparent adverse reactions. While there are no reported cases of leukemogenesis in these trials, clonal expansion of cells with integration in the *HMGA2* gene was observed in a single patient (Cavazzana-Calvo et al., 2010). However, a subsequent study of 22 patients do not observe any clonal expansion (Thompson et al., 2018).

Other retroviruses have been proposed for use in human gene therapy including ASLV-based vectors (Hu et al., 2007). Transgenic mouse lines have been developed using an ASLV-based retroviral vector system for delivery of genes to preimplantation mouse embryos (Federspiel et al., 1994; Federspiel et al., 1996). In other studies, these vectors were shown to transduce rhesus macaque CD34^+^ hematopoietic progenitor cells efficiently (33%) and stably up to 18 months (Hu et al., 2007). In a study looking at a limited number of integration sites of rhesus long-term repopulating cells there was no detectable integration at enhancers, promoters, or oncogenes (Hu et al., 2008). This integration pattern could be linked to ASLV IN interactions with the FACT complex, which is proposed to be enriched in gene bodies, (Winans et al., 2017). To date ASLV vectors have not progressed to human gene therapy trials.

Foamy viruses (FV), such as PFV, are not known to cause disease in animal infections or xenotropic human infections. The FV life cycle differs from other retroviruses and lentiviruses which precludes them from being efficiently pseudotyped (reviewed in (Lindemann and Rethwilm, 2011)). However, it is possible to generate high titer FV vectors which have been shown to transduce several cell types including human primary macrophages, human and rhesus embryonic stem cells, human induced pluripotent stem cells, and murine hematopoietic stem cells (Vassilopoulos et al., 2001; Trobridge et al., 2002a; Gharwan et al., 2007; Taylor et al., 2008; Deyle et al., 2013; Nasimuzzaman et al., 2016; Rajawat et al., 2019). FVs are not known to have a host integration co-factor and have little preference for genomic features. Sequencing FV integration sites suggests that FV integration shows a slight preference for TSSs and CpG islands, but less so than MLV integration (Nowrouzi et al., 2006; Trobridge et al., 2006; Serrao et al., 2015). Importantly, FV integration does not appear to be oncogenic. Five dogs with canine leukocyte adhesion deficiency (CLAD) caused by deficient expression of CD18 were treated with a FV gene therapy vector (Bauer et al., 2008; Ohmine et al., 2011; Bauer et al., 2013). Autologous CD34^+^ hematopoietic stem cells were transduced and infused to the animals (Bauer et al., 2008). Four of the dogs were cured of CLAD, a phenotype which lasted 4–7 years (Bauer et al., 2013). There was no evidence of leukemia in any of these animals (Ohmine et al., 2011; Bauer et al., 2013). Similar FV gene therapy of myoblast cells *ex vivo* followed by intramuscular transplantation has successfully led to muscle regeneration in a mouse model of Duchenne muscular dystrophy (Meng et al., 2020).

While most retroviral gene therapy protocols include transduction of cells *ex vivo*, FV has also been directly administered intravenously to dogs (Burtner et al., 2014; Humbert et al., 2018). Six dogs with X-SCID were given FV vectors expressing the *γc* gene intravenously. The FV vectors expressed the natural Env and were not targeted to a particular cell type. However, two animals were treated with granulocyte colony-stimulating factor (G-CSF) and AMD3100 to mobilize hematopoietic stem and progenitor cells, followed by injection of the FV gene therapy vector, and showed the greatest survival of 2.5 years (Burtner et al., 2014). There was no evidence of oncogenesis in any of the animals. These studies suggest that FV vectors may naturally present the least possibility of oncogenic transformation with the benefit of *in vivo* delivery. The greatest limitation is the inability to pseudotype and alter the tropism of FV vectors.

### Integrase Fusions

Attempts to target retroviral integration to a sequence specific site with chimeric IN proteins were reported over 25 years ago (Bushman, 1994; Kniazhanskaia et al., 2011). It was recognized that retroviral integration was possible at multiple sites throughout a host genome with some preference for particular sites (Vijaya et al., 1986; Shih et al., 1988). At that time, it was known that the *Saccharomyces cerevisiae* LTR retrotransposon Ty3 precisely integrates at the transcription start site of tRNA genes (Chalker and Sandmeyer, 1992). It was suggested that Ty3 IN might bind to polymerase III associated transcription factors which directed integration to tRNA genes. Several groups investigated the possibility of directing retroviral integration to specific genomic sites by engineering a chimera of a DNA binding domain (DBD) and IN. The DNA binding domain (DBD) of lambda repressor was fused to the HIV-1 IN amino terminus or the *E. coli* LexA repressor full length protein or its DBD was fused to the HIV-1 IN carboxyl terminus (Bushman, 1994; Goulaouic and Chow, 1996). Two chimeras of the ASLV IN were engineered with the LexA DBD at either the amino or carboxyl termini (Katz, Merkel et al., 1996). The LexA recognition site is 16 bp and the lambda repressor binds 17 bp (Lewis et al., 1994; Albright and Matthews, 1998). Recombinant chimeric proteins were purified and their abilities to target integration to a specific DNA site were assayed *in vitro*. While the results were not quantified, these experiments suggested that chimeric INs showed an increase of integration efficiency near a DNA sequence specific site under *in vitro* conditions. These early experiments were highly suggestive that retroviral INs were amenable to sequence specific targeting, and might be enhanced with further development. Both LexA and lambda repressor must dimerize to bind DNA, which may have complicated these experiments.

Zinc finger DNA binding proteins are capable of binding specific sequences as monomers. These proteins consist of zinc finger domains that individually bind a specific 3 bp sequence. The murine Zif268/Egr1 transcription factor has 3 zinc fingers and recognizes a 9 bp sequence (Pavletich and Pabo, 1991). Zif268 was fused to the carboxyl terminus of HIV-1 IN and tested for activity in cellular integration assays (Bushman and Miller, 1997). HIV-1 viruses with the IN-Zif268 fusion could not be produced by transfection of HEK293T cells. However, virion production was rescued by generating virus particles with a mixture of wild type and chimeric IN. The HEK293T producer cells were transfected with varying ratios of HIV-1 plasmid encoding wild type IN or IN-Zif268. These virions were added to target cells and PICs were obtained 7 h post infection. The PICs were allowed to integrate to a target *in vitro* and integration sites were evaluated by high resolution gel electrophoresis. PICs with the chimeric IN displayed some preference for the Zif268 binding site, while also integrating at multiple other sites. Whether the integration sites of the cellular infection also occurred near Zif268 binding sites is unknown.

A more definitive strategy to engineer HIV-1 viruses including the chimeric IN protein employed delivery *in trans* (Holmes-Son and Chow, 2000). IN chimeras with carboxyl terminal full length LexA protein or its DBD were cloned to the 3′ end of *vpr*. An HIV-1 protease cleavage site between Vpr and IN-LexA allowed the chimera to be packaged in the virion via Vpr targeting and subsequently liberated by HIV-1 protease cleavage within the virion. The IN encoded by *pol* was engineered to be catalytically inactive. This strategy would ensure that catalytically active integration complexes must include the chimeric IN. The production by HEK293T cells of virions with Vpr-IN fusions was significantly reduced compared to Vpr alone. When equivalent amounts of virions were added to target cells, the fusion of IN-LexA reduced integration efficiency but was readily detectable. Reduced integration efficiency may be an expected consequence of the restrictions imposed by targeting integration *in vivo* to a limited number of sites. However, sequencing a limited number of the integration sites from these IN-LexA infected cells found that LexA recognition sites were not observed within 200–300 bp (Holmes-Son and Chow, 2002).

A synthetic protein of 6 zinc finger domains termed E2C binds specifically to an 18 bp sequence. This protein was fused to the amino or carboxyl terminus of HIV-1 IN and analyzed for integration to a plasmid encoding the E2C recognition site (Tan et al., 2004). Although the assays were not quantitated, the chimeras displayed a dramatic preference for integration within 20 bp of the E2C binding site. A caveat to this apparent targeting is the use of a PCR-based assay for integration that does not distinguish between the joining of two viral DNA ends mimicking integration *in vivo* vs. a non-physiological joining of a single viral DNA end. The E2C chimeras were also assayed for integration during cellular infection using the Vpr fusion strategy developed with the LexA chimeras (Tan et al., 2006). The E2C recognition site is present in the human genome in the *erbB-2* gene 5′ untranslated region. Viruses with the E2C chimeras displayed 11–24% infection efficiency compared to wild type virus. Quantitative PCR allowed the measurement of the total number of integrated proviruses and the number of proviruses near the E2C site. While 0.15% of wild type HIV-1 proviruses were near the E2C site, 1.5% of viruses with E2C at the amino terminus of IN integrated near the recognition site (Tan et al., 2006).

Similar to the increased targeting observed with an E2C chimera, a limited increase in targeting HIV-1 integration was observed with an IN fusion to I-PpoI (Schenkwein et al., 2013). I-Ppol, a slime mold homing endonuclease, recognizes a 15 bp sequence that is present in eukaryotic rDNA at ∼600 copies/genome. An enzymatically inactive mutant of I-PpoI was fused to the carboxyl terminus of HIV-1 IN (Schenkwein et al., 2010). Lentiviral vector particles were produced with a mixture of catalytically inactive HIV-1 IN and the IN-I-PpoI chimera. Sequencing integration sites revealed that 2.7% of integration sites with the chimera were at rDNA loci while only 0.1% of wild type HIV-1 IN integration sites were at these sites. The targeting by this chimera may have been confounded by the dimerization of I-PpoI.

In the past several years multiple retroviral intasomes have been visualized. These integration complexes include tetramers (PFV, HTLV-1), octamers (MMTV, RSV), and hexadecamer (maedi visna virus, MVV) (Hare et al., 2010; Maertens et al., 2010; Ballandras-Colas et al., 2016; Yin et al., 2016; Ballandras-Colas et al., 2017; Barski et al., 2020; Bhatt et al., 2020). Several multimeric forms have been observed for simian immunodeficiency virus (SIV) and HIV-1 IN (Passos et al., 2017; Cook et al., 2020). The multimerization of INs suggest that fusion of DNA binding domains to IN may negatively affect the assembly of functional complexes. The most promising approach for an IN fusion is possibly the tetrameric PFV intasome that does not appear to require a host co-factor. An elegant pairing of point mutations has been shown to direct PFV IN monomers to the catalytically active “inner” positions or the structural “outer” positions of the intasome (Maskell et al., 2015). While the inner protomers make extensive DNA and protein contacts, the amino and carboxyl termini of the outer protomers are unstructured and may readily tolerate the addition of DNA or chromatin binding domains. The recent visualization of retroviral intasome multimeric structures provide clarifying insights into the practicality of engineering functional IN fusions for targeting integration.

### Fusions of Intasome Tethering Factors

Retroviral integration site selection may be redirected by altering the chromatin binding domain of the respective cellular co-factors. Perhaps the best studied integration cofactor is LEDGF/p75. There have been a variety of reported alternative chromatin binding domains fused to the IBD of LEDGF/p75. The first successful attempt involved fusing the first 31 amino acids of kaposi’s sarcoma-associated herpesvirus (KSHV) latency-associated nuclear antigen (LANA) to LEDGF/p75 (93–530) lacking the PWWP domain (Meehan et al., 2009). KSHV is a gammaherpes virus whose genome persists as a DNA episome via the N-terminal residues (5–13) of LANA which interact with the groove between histones 2A and 2B (Chang et al., 1994; Ballestas et al., 1999; Barbera et al., 2006). While integration site selection was not examined in this study, expression of this fusion in LEDGF/p75 deletion cells led to rescued HIV-1 infectivity (Meehan et al., 2009). Other protein domains such as plant homeodomain (PHD) finger from inhibitor of growth protein 2 (ING2) and the chromodomain from heterochromatin binding protein 1alpha (HP1alpha or CBX5) have also been fused to the LEDGF IBD (Ferris et al., 2010; Silvers et al., 2010). The PHD domain binds H3K4me3, typically found in regions of active transcription, and the HP1alpha chromodomain binds H3K9me2/3 located in heterochromatin (Vakoc et al., 2005; Wysocka et al., 2006; Ruthenburg et al., 2007). These fusion proteins were expressed in a LEDGF/p75 deletion cell line. In these cases, HIV-1 integration was redirected toward TSSs and actively expressed genes and regions of lower gene expression, respectively. A second study using HP1alpha saw a similar trend with retargeting toward repetitive sequences and away from genes, typical of heterochromatin (Silvers et al., 2010). An alternative approach used fusions of heterochromatin protein 1beta (also called chromobox protein homolog 1 or CBX1) to IBD which retargeted integration to silent gene regions (Maison and Almouzni, 2004; Gijsbers et al., 2010). Interestingly, a marker gene was efficiently expressed despite residing in transcriptionally silent chromatin.

Most of the re-targeting studies of LEDGF/p75 fusions have been performed in LEDGF/p75 knockdown or depleted cells. This is an impractical approach for patients due to the necessity of removing endogenous LEDGF/p75 prior to gene therapy. There is one reported attempt of using a LEDGF/p75 fusion in a wild type background (Vets et al., 2013). Cells were electroporated to introduce mRNA encoding LEDGF/p75 IBD with an amino terminal fusion of CBX1. The following day the cells were transduced with an HIV-1 vector. Interestingly, integration to RefSeq genes was reduced from 75.2 to 54.1%. Similar to other studies, short-term expression of a transgene from the provirus was efficient but long-term expression is unknown. While it may be possible to target integration to safe harbor heterochromatin regions, the long-term expression of the transgene is unclear. Indeed, retroviruses such as ASLV, which has a preference for integration into heterochromatin, may be silenced over time and long-term expression is dependent on being in transcriptionally active regions (Senigl et al., 2017; Miklik et al., 2018). This gene silencing can be counteracted by the incorporation of an anti-silencing CpG island core sequence in the provirus (Senigl et al., 2017). However, the potential consequences of a CpG island in an integrated provirus in patients are unclear.

### Tether Independent Integration Targeting

PFV IN is not known to require a host co-factor that tethers integration complexes to chromatin. However, the PFV IN CTD appears to interact with the amino terminus of nucleosome protein H2A (Maskell et al., 2015). In addition, a three amino acid motif in the carboxyl terminus of PFV Gag also appears to interact with histones H2A and H2B (Tobaly-Tapiero et al., 2008; Hocum et al., 2016). It is not clear what role histone PTMs might have on this interaction. It has been shown that alanine mutations of the Gag chromatin binding site (CBS) alter the integration site selection in cells away from TSSs and CpG islands (Hocum et al., 2016). Combination of the Gag CBS alanine mutant with a PFV IN fusion to the CBX1 protein had little further effect on re-targeting integration to any chromatin element (Hocum et al., 2016). Perhaps alternative PFV IN targeting fusions will prove better able to direct integration in the context of the Gag CBS mutation.

Another avenue for tether independent targeting is to generate retroviral vectors that no longer need their respective co-factor. This is not possible with HIV-1 IN as LEDGF/p75 not only plays a critical role in tethering but is also essential for IN catalytic activity (Llano et al., 2004; Cherepanov et al., 2005; Llano et al., 2006b; Vandekerckhove et al., 2006; Zielske and Stevenson, 2006; McKee et al., 2008; Kessl et al., 2011). However, a different situation exists for the generation of MLV-based retroviral vectors which are BET protein independent. While important for integration site selection, the carboxyl terminal tail of MLV IN is not essential for catalytic activity *in vitro* or infection of cells (Aiyer et al., 2014; Larue et al., 2014). Biochemical studies have confirmed that these truncations abolish interaction with the BET proteins, demonstrating its significance for the binding interface (Larue et al., 2014). Truncations or mutations in the carboxyl terminal tail of MLV IN reduce MLV integration near TSSs without significant effects on replication (Aiyer et al., 2014; El Ashkar et al., 2014). Studies using alanine mutagenesis of the carboxyl terminal tail, deletion of the tail, or treatment with JQ-1 all led to a decrease in MLV integration at TSSs (Sharma et al., 2013; Aiyer et al., 2014; El Ashkar et al., 2014). These studies demonstrate that MLV-based vectors can be guided away from TSSs by removal of interaction with BET proteins. Importantly, mutations of the MLV carboxyl terminal tail is not sufficient to retarget all integration away from TSSs, raising concerns that this avenue would not be appropriate for gene therapy.

### Remaining Questions and Conclusions

The first goal of safer retroviral gene therapy vectors is to remove the possibility of cellular transformation and oncogenesis. This must be coupled with sufficient functional rescue and sustained gene expression. Recent evidence suggests that lentiviral-based gene therapy vectors are closer to achieving these goals (Hocum et al., 2016; El Ashkar et al., 2017). Functional rescue of monogenic disorders has been reported with integration in gene silent regions (Vets et al., 2013). However, it is unclear if such retargeting will allow for long-term expression due to either limited experimental time points or the recent initiation of the gene therapy trials (Senigl et al., 2017; Miklik et al., 2018). Using methods such as alteration to retroviral IN or host cofactors remain untested in patients. Recent studies of MLV and HIV-1 retroviral vectors with altered IN targeting suggest that the constraints imposed by host tethering factors may be reduced but not eliminated (Hocum et al., 2016; El Ashkar et al., 2017). Integration site analysis of BET independent MLV infection of a MYC/Runx2 mouse model revealed less integration at TSSs, decreased rate of tumorigenesis, and decreased integration at histone marks associated with BET proteins (Loyola et al., 2019). However, in the mice that developed tumors, integration was in regions containing oncogenic genes. This implies that residual MLV integration into TSSs can still lead to oncogenesis. For this reason, it appears that MLV-based vectors may not be clinically relevant without significant additional modification. Non-integrating lentiviral vectors with enzymatic mutations of integrase are in development but have not yet entered clinical use (reviewed in (Luis, 2020)).

One question in retroviral gene therapy is to what extent will directed integration be possible: sequence specific sites, unique histone PTMs, or genomic regions? Retroviral INs may impose some subtle preference for sequence at the integration site, but the preference is not stringent (Bennett et al., 2014). Directing retroviral vector integration to highly repetitive genomic sequences could be a first step toward advancing integration sequence specific targeting. The probability targeting a single site using retroviral-based vectors is empirically unlikely; a single PIC would have to search the entire genome to find the select site. This would lead to a dramatic reduction of transduction efficiency. Sequence site specific targeting by Clustered Regularly Interspaced Short Palindromic Repeats (CRISPR), transcription activator-like effector nucleases (TALENs), or zinc finger nucleases (ZFNs) is successful due to their effective concentration in the nucleus, allowing for genome-wide searching (Romero et al., 2018). These genome editors are either transfected or transduced into cells allowing for high protein expression and multiple nuclear complexes to search the genome. Although cultured cells may be transduced with multiplicities of infection >1, it seems unlikely that sufficient numbers of retroviruses to perform sequence specific targeting could efficiently infect a single cell. More optimistic re-directing of retroviral integration may be to repetitive sequences in safe harbors or to abundant histone PTMs that mark such regions.
